# The Evolution and Recent Advances in Diagnostic Criteria for Idiopathic Multicentric Castleman Disease

**DOI:** 10.1002/ajh.70039

**Published:** 2025-08-21

**Authors:** FNU Alnoor, Nicholas C. Spies, Jyoti Kumar, Peyman Samghabadi, Oscar Silva, Matt X. Luo, Karen M. Chisholm, Jingjing Zhang, Alexandra Rangel, David Ng, Peng Li, Robert S. Ohgami

**Affiliations:** ^1^ Department of Pathology and Laboratory Medicine University of Miami Miller School of Medicine Miami Florida USA; ^2^ ARUP Laboratories, Department of Pathology University of Utah Salt Lake City Utah USA; ^3^ Division of Pathology, Department of Diagnostic Medicine The University of Texas at Austin Austin Texas USA; ^4^ Department of Pathology University of California San Francisco California USA; ^5^ Department of Pathology Stanford University Stanford California USA; ^6^ Department of Laboratory Medicine and Pathology University of Washington Seattle Washington USA; ^7^ Department of Laboratories Seattle Children's Hospital Seattle Washington USA; ^8^ University of Colorado Anschutz Medical Campus Aurora Colorado USA

**Keywords:** Castleman disease, fibroblastic reticular cells, follicular dendritic cells, idiopathic multicentric Castleman disease, IgG4‐related disease, interleukin‐6

## Abstract

Idiopathic multicentric Castleman disease (iMCD) is a rare cytokine‐driven disorder characterized by systemic inflammation, organ dysfunction, and altered lymph node microscopic architecture. Over the past decade, diagnostic criteria have evolved significantly, integrating clinical, histopathological, and molecular biomarker advancements. Key drivers of iMCD pathogenesis, such as interleukin‐6 dysregulation and other dysfunctional cytokine signaling, have been identified and led to the development of targeted therapies like siltuximab and tocilizumab. Histopathologic refinements have highlighted distinct subtypes, such as hypervascular, plasmacytic, and mixed histologic patterns, while molecular discoveries have unveiled potential paraneoplastic and clonal processes. Emerging technologies, including single‐cell sequencing, spatial transcriptomics, and digital pathology, offer promise in refining diagnostic precision and advancing personalized medicine. This review synthesizes historical frameworks, recent breakthroughs, and future directions in iMCD diagnostics, emphasizing the importance of multidisciplinary approaches for improved patient outcomes.

## Graphical abstract

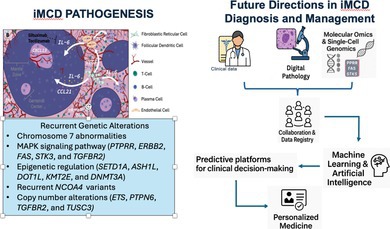

## 1 Introduction

Castleman disease (CD) was first described in the 1950s by Dr. Benjamin Castleman and colleagues as an unusual form of benign lymph node hyperplasia, establishing the entity now known as unicentric Castleman disease (UCD) [1, 2]. Subsequent cases involving multiple lymph nodes led to the classification of idiopathic multicentric Castleman disease (iMCD), a rare cytokine‐driven disorder characterized by systemic inflammation and widespread lymphadenopathy [3]. Unlike HHV‐8‐associated MCD, iMCD has no known viral etiology [4]. Patients often present with nonspecific symptoms such as fever, weight loss, and lymphadenopathy, mimicking other inflammatory, infectious, or neoplastic conditions.

Recent advancements, driven by collaborative efforts such as the Castleman Disease Collaborative Network (CDCN), have established standardized diagnostic criteria integrating clinical, histopathologic, and molecular findings [3]. Key insights include the pivotal role of interleukin‐6 (IL‐6) in driving disease pathology and the efficacy of targeted anti‐IL‐6 therapies such as siltuximab and tocilizumab [5]. Histopathologic refinements now emphasize clinicopathologic correlations, moving beyond traditional hypervascular and plasmacytic subtypes [6].

Emerging tools, including single‐cell sequencing and spatial transcriptomics, offer new opportunities to unravel the cellular and molecular underpinnings of iMCD, improve diagnostic precision, stratify subtypes, and identify novel therapeutic targets [7]. This review summarizes the evolution of iMCD diagnostics, highlighting key advances and future directions to improve patient outcomes through multidisciplinary and personalized approaches.

## 2 Materials and Methods

### 2.1 Literature Review

A literature search for articles and studies related to iMCD was performed using PubMed and Scopus to identify articles published between 1954 and 2025. Keywords used included “Castleman disease,” “Castleman's disease,” “angiofollicular lymph node hyperplasia,” “giant lymph node hyperplasia,” and “idiopathic multicentric Castleman disease.” Articles were evaluated for inclusion if they discussed clinical, histopathological, molecular, new technological, or therapeutic advances related to iMCD (Figure S1 and Table S1). This study was approved by the Institutional Review Board (IRB).

### 2.2 Pathology Panel

The iMCD Pathology Working Group was formed to guide the review process. Panelists were selected based on their prior contributions to iMCD research, diagnostic criteria, peer‐reviewed publications, and potential for ongoing impact in iMCD research. Eleven members were invited from leading academic and research institutions across the United States to ensure diverse expertise.

### 2.3 Consensus Development

The iMCD Pathology Working Group convened remotely in November 2024 for an initial meeting to outline the project's scope. The review underwent four rounds of iterative feedback and revision to ensure accuracy and comprehensive coverage. Recommendations from the CDCN and international diagnostic guidelines were incorporated to align findings with current consensus. This collaborative process aimed to produce a thorough and evidence‐based analysis of diagnostic advancements in iMCD. No new datasets were generated or analyzed during the current study.

## 3 Results

### 3.1 The Early Evolution of Histopathologic Criteria in CD

**FIGURE 1:**
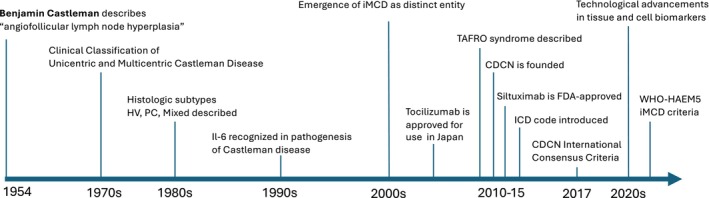
Historical perspective of idiopathic multicentric Castleman disease. CDCN, Castleman's Disease Collaborative Network; FDA, Food and Drug Administration; HV, hypervascular type; iMCD, idiopathic multicentric Castleman disease; PC, plasma cell type; TAFRO, thrombocytopenia, anasarca, fever, reticulin fibrosis (or renal dysfunction), and organomegaly; WHO‐HAEM5, World Health Organization Classification of Haematolymphoid Tumors, 5th Edition. [Color figure can be viewed at wileyonlinelibrary.com]

**FIGURE 2:**
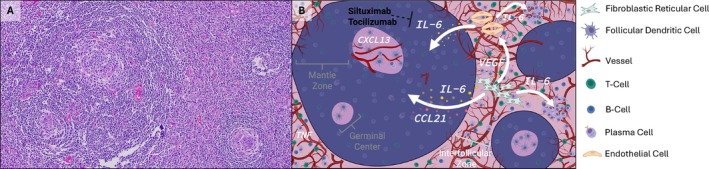
Histopathology and Integrative schematic of the lymph node microenvironment in idiopathic multicentric Castleman disease (iMCD). (A) H&E‐stained lymph node from a patient with iMCD showing regressed germinal centers surrounded by concentric mantle zone B cells, producing the characteristic “onion‐skin” pattern. On the left side of the H&E image, two adjacent atretic germinal centers are encircled by a coalesced mantle zone, forming a “twinned” follicle structure. On the bottom right a single follicle with a regressed germinal center and follicular dendritic cell predominance is seen. (B) Schematic depiction of key histologic compartments and cellular interactions in iMCD. A germinal center (GC), mantle zone, and the interfollicular zones are outlined. Fibroblastic reticular cells (FRCs) and endothelial cells are shown as major sources of IL‐6 (indicated by white arrows), which promotes systemic inflammation and stimulates plasma cell proliferation. FRCs also secrete CCL21, and VEGF is depicted near vascular structures, contributing to angiogenesis and vascular leak. CXCL13, a B‐cell attracting chemokine, is highlighted in the GC region. Therapeutic targeting agents such as siltuximab (anti‐IL‐6) and tocilizumab (anti‐IL‐6R) are labeled near the IL‐6 pathway. The schematic integrates spatial histology with cytokine‐mediated mechanisms driving disease pathogenesis. A key of the cells and structures in the depiction is seen on the right side. [Color figure can be viewed at wileyonlinelibrary.com]

The diagnostic framework for Castleman disease (CD) has evolved from rigid histologic subtyping to an integrated clinicopathologic approach (Figure 1). First described by Castleman et al. in 1954–1956, early CD cases were typically characterized by a single enlarged lymph node with regressed germinal centers, capillary proliferation, and hyalinization (Figure 2) [1, 2]. This pattern later became known as the hyaline vascular (HV) subtype of CD. In the 1970s, a second histopathologic variant was recognized. Keller et al. described a plasma cell (PC) type, marked by interfollicular sheets of mature polytypic PCs [8]. Patients with the PC type often showed more inflammation and systemic symptoms. Mixed HV/PC patterns were also noted, though without strict criteria. By the late 1970s, CD was seen as a distinct entity with HV and PC types.

In the 1980s, Japanese and US clinicians described multi‐nodal CD cases, initially termed “idiopathic plasmacytic lymphadenopathy with polyclonal hyperimmunoglobulinemia” (IPL), later redefined as multicentric CD (MCD) [9, 10, 11]. MCD featured multicentric nodal involvement, systemic inflammation, and PC or mixed histopathology. Then, in the 1980s to 1990s, MCD in HIV/AIDS patients was linked to Kaposi sarcoma [12, 13, 14, 15]. This led to formal recognition of “HHV‐8‐associated multicentric Castleman disease” as a distinct subtype, diagnosed via histology and HHV‐8 detection; however, not all MCD patients had an HHV‐8 infection. Some HHV‐8‐negative MCD cases were later found to have polyneuropathy, organomegaly, endocrinopathy, monoclonal protein, skin changes (POEMS) syndrome, driven by clonal PCs [16].

Still, some MCD patients lacked both HHV‐8 and POEMS; truly “idiopathic” [9, 11]. However, iMCD was not fully recognized until the 2000s [4]. iMCD showed characteristic lymph node histology, severe systemic inflammation, and IL‐6 was found to be a key mediator, which led to trials of anti‐IL‐6 therapies (e.g., siltuximab and tocilizumab). Then, in the early 2000s, a clinically severe and rapidly progressive iMCD subtype, TAFRO, was first described (*T*hrombocytopenia, *A*nasarca, *F*ever, *R*eticulin fibrosis or *R*enal dysfunction, and *O*rganomegaly) [17, 18, 19, 20].

### 3.2 Current Diagnostic and Histopathologic Grading for iMCD

CD, especially iMCD, lacked standardized diagnostic criteria for years. However, in 2017, the CDCN led the development of the first evidence‐based iMCD diagnostic criteria [3]. A panel of 34 international experts reviewed hundreds of cases and published consensus criteria now refined and adopted by the fifth edition of the World Health Organization's (WHO) Classification of Haematolymphoid Tumors (WHO‐HAEM5) [3, 21, 22].

According to the WHO, diagnosis requires three major criteria and 2 of 11 minor clinical/lab criteria (Table 1). Major criteria include (1) multicentric lymphadenopathy, (2) CD HV, PC, or mixed type histology, and (3) negative HHV‐8 Latency‐Associated Nuclear Antigen (LANA) by immunohistochemistry (IHC). Histomorphologic grading (on a scale of 0–3) is required and evaluates germinal center regression, follicular dendritic cell prominence, vascularity, plasmacytosis, and germinal center hyperplasia (Table 2) [3]. Minor criteria require ≥ 2 of 11 findings, including ≥ 1 abnormal lab marker (e.g., anemia, elevated CRP). Abnormal clinical signs include hepatosplenomegaly, constitutional symptoms, skin lesions, effusions/edema, and lung involvement with lymphocytic interstitial pneumonitis. Finally, mimics of iMCD (e.g., autoimmune, infectious, neoplastic) must be excluded. The 2017 study showed that patients essentially not meeting these criteria failed to respond to siltuximab, confirming diagnostic relevance [3]. We do note that in the original 2017 Fajgenbaum et al. diagnostic criteria, there were two major criteria, which included multicentric lymphadenopathy and CD histology, but ruling out HHV‐8 infection was included only in exclusionary criteria and not in major criteria [3].

**TABLE 1:** Diagnostic criteria for iMCD.^a^

Criteria	iMCD‐NOS	iMCD‐TAFRO
Major criteria (3 of 3 required)		
Lymph node enlargement	≥ 2 lymph node stations (> 10 mm short axis)	≥ 2 lymph node stations (≤ 10 mm)
Morphological features	Grades 2–3 regressed follicles OR Grades 2–3 plasmacytosis	Grades 2–3 regressed follicles OR Grades 2–3 plasmacytosis
KSHV/HHV‐8 LANA IHC	Negative	Negative
Additional TAFRO‐specific major criteria	Not applicable	All five required: Anasarca: pleural effusions, ascites, subcutaneous edema Thrombocytopenia: < 10 × 10^4^/μL Fever/Inflammation: > 37.5°C AND/OR CRP ≥ 2 mg/dL Organomegaly: lymphadenopathy in > 2 stations AND/OR hepatomegaly AND/OR splenomegaly Either: Bone marrow reticulin fibrosis/megakaryocytic hyperplasia OR Renal insufficiency: eGFR < 60 mL/min/1.73 m^2^ OR Creatinine > 1.3 mg/dL (males), > 1.1 mg/dL (females) OR Renal failure requiring dialysis
Minor criteria	Minimum 2 required (≥ 1 must be laboratory)	Not applicable if TAFRO criteria met
Laboratory criteria	Anemia: Hb < 12.5 g/dL (males), < 11.5 g/dL (females) Platelets: < 150 k/μL OR > 400 k/μL CRP > 1 mg/dL or ESR > 15 mm/h Renal dysfunction (eGFR < 60 mL/min/1.73 m^2^) or proteinuria (total protein 150 mg/24 h or 10 mg/100 mL) Polyclonal hypergammaglobulinemia (total γ globulin or IgG > 1700 mg/dL)	
Clinical criteria	Constitutional symptoms (documented fever, night sweats, weight loss) Hepatomegaly and/or splenomegaly (radiologically confirmed) Fluid accumulation (edema, pleural effusions, ascites) Eruptive cherry hemangiomatosis or violaceous papules Lymphocytic interstitial pneumonitis (biopsy‐proven)	
Laboratory supportive features (not required; may be present)	IL‐6 elevation (> 2× upper limit of normal) VEGF elevation Elevated alkaline phosphatase without elevated bilirubin/transaminases β2‐microglobulin elevation	IL‐6 elevation (> 2× upper limit of normal) VEGF elevation IgA, IgE elevation LDH elevation β2‐microglobulin elevation
Histopathological features (not required; may be present)	Lymph node > 10 mm (short axis) Mixed/plasmacytic features	Grades 2–3 vascularity Lymph nodes typically ≤ 10 mm Grades 2–3 hyperplastic germinal centers Reticulin marrow fibrosis
Exclusion criteria (must be ruled out)		
Infections	KSHV/HHV‐8 (positive LANA) EBV (infectious mononucleosis or chronic active EBV) COVID‐19 (active) Uncontrolled infections, for example: ○ Active tuberculosis ○ Cytomegalovirus ○ Human Immunodeficiency Virus ○ Toxoplasmosis	
Autoimmune, autoinflammatory diseases	SLE (meets ACR criteria) Rheumatoid arthritis (meets ACR criteria) Adult‐onset Still disease Juvenile idiopathic arthritis Sjögren syndrome ALPS IgG4‐related disease	
Malignancies	Lymphoma Plasma cell myeloma/plasmacytoma POEMS syndrome Follicular dendritic cell sarcoma	

Abbreviations: ACR, American College of Rheumatology; ALPS, autoimmune lymphoproliferative syndrome; CRP, C‐reactive protein; eGFR, estimated glomerular filtration rate; HHV‐8, human herpesvirus 8; IHC, immunohistochemistry; KSHV, Kaposi sarcoma herpesvirus; LANA, latency‐associated nuclear antigen; POEMS, polyneuropathy, organomegaly, endocrinopathy, M‐protein, skin changes; SLE, systemic lupus erythematosus; VEGF, vascular endothelial growth factor.

^a^ All laboratory values should be interpreted in clinical context. Some patients may have excluded conditions concurrent with Castleman disease; careful clinical evaluation is necessary. Diagnostic workup should include complete blood count, comprehensive metabolic panel, inflammatory markers, and appropriate imaging studies.

**TABLE 2:** Castleman disease histologic grading.

Histologic features	Grading			
	0	1	2	3
Regressed germinal centers	Essentially Absent	Few (10%–< 25% of all follicles)	Many (25%–< 50% of all follicles)	Most (≥ 50% of all follicles)
Follicular dendritic cell (FDC) prominence	Not prominent	Mild (10%–< 25% of cellularity)	Moderate (25%–< 50% of cellularity)	Very prominent (≥ 50% of cellularity)
Vascularity (interfollicular)	Normal	Mildly increased (10%–< 25% of cellularity)	Moderately increased (25%–< 50% of cellularity)	Very prominent (≥ 50% of cellularity)
Hyperplastic geminal centers	None	Few (10%–< 25% of all follicles)	Many (25%–< 50% of all follicles)	Most (≥ 50% of all follicles)
Plasmacytosis	Normal	Mildly increased (10%–< 25% of cellularity)	Moderately increased (25%–< 50% of cellularity)	Marked increased (≥ 50% of cellularity)

Within iMCD, two subtypes are additionally now recognized: iMCD‐TAFRO and iMCD‐NOS. iMCD‐TAFRO lymph nodes show vascularity, and atrophic follicles are more prominent than in iMCD‐not otherwise specified (NOS). In iMCD‐TAFRO, bone marrow biopsies often show hypercellularity, megakaryocytic hyperplasia, and reticulin fibrosis, supporting the diagnosis and correlating with the thrombocytopenia observed in TAFRO syndrome [23, 24]. Imaging supports the diagnosis, often revealing pleural effusions, ascites, hepatosplenomegaly, and systemic edema. Due to its rapid course, iMCD‐TAFRO needs aggressive treatment, IL‐6 blockade (siltuximab or tocilizumab), corticosteroids, and immunosuppressive therapy such as cyclosporine.

## 4 Biomarker Discoveries and Genetic Insights

### 4.1 Biomarkers

Despite defined criteria, no single laboratory biomarker is pathognomonic for iMCD. Individual laboratory biomarkers for iMCD diagnosis are nonspecific and include elevated IL‐6, CRP, ESR, VEGF, chemokine (C‐X‐C motif) ligand 13 (CXCL13), IL‐10, and soluble interleukin‐2 receptor (sIL‐2R) [3]. Additional findings include hypoalbuminemia, polyclonal hypergammaglobulinemia (particularly IgG or IgA), elevated free light chains, and evidence of renal dysfunction or proteinuria [4]. IL‐6 plays a central role in iMCD pathogenesis, driving many of its hallmark features, including systemic inflammation, fever, anemia, and hypergammaglobulinemia. Elevated IL‐6 levels stimulate hepatic production of acute‐phase reactants such as CRP and contribute to B‐cell activation and recruitment and VEGF overproduction (Figure 2). Correspondingly, blocking IL‐6 has profound therapeutic effects. Monoclonal antibodies such as siltuximab (anti‐IL‐6) or tocilizumab (anti‐IL‐6 receptor) have demonstrated significant efficacy in many patients, highlighting the cytokine's pivotal role in disease pathology [5, 25, 26]. Tocilizumab was the first such agent used in iMCD, with sustained efficacy shown in both trials and real‐world studies [27]. Siltuximab is currently the only FDA‐approved therapy for iMCD, based on an international randomized trial where 34% of iMCD patients achieved objective response to siltuximab (with or without steroids), underscoring the heterogeneity of pathogenic mechanisms in iMCD [28].

Given that nearly half of iMCD patients may not respond to IL‐6 blockade, alternative therapeutic approaches have been explored. One such strategy is a multi‐agent immunomodulatory regimen referred to as thalidomide, cyclophosphamide, and prednisone (TCP). In a Phase 2 trial of newly diagnosed iMCD, an oral TCP regimen achieved approximately 50% durable tumor and symptomatic response [29]. This study demonstrated significant improvements in symptoms and inflammatory markers in responders, with an overall 1‐year progression‐free survival of 60%. The TCP regimen is thought to work by targeting multiple pathogenic pathways. Thalidomide has immunomodulatory and antiangiogenic effects and reduces TNF and VEGF, as well as inhibiting PC activity; cyclophosphamide is cytotoxic to proliferating lymphoid cells; and prednisone broadly dampens the inflammatory cytokine storm. This regimen was relatively well tolerated in the trial and has been used in resource‐limited settings where some therapies are unavailable, providing a proof of concept that non‐IL‐6 targeted therapies can induce remission in iMCD.

Elevations of other cytokines and markers in iMCD are also noteworthy. Increased CXCL13, a chemokine involved in B‐cell recruitment and germinal center formation, supports enhanced lymphoid activity. IL‐10, an anti‐inflammatory cytokine, exacerbates immune dysregulation by suppressing normal immune responses and promoting polytypic PC proliferation. Elevated sIL‐2R indicates aberrant T‐cell activation, which may contribute to the inflammatory environment characteristic of iMCD [5, 25, 26].

The complex and nonspecific nature of these markers highlights the intricate interplay of B‐cell and T‐cell activation in iMCD pathogenesis, underscoring their potential for disease monitoring and targeted therapy [19, 30]. Efforts to develop multi‐marker panels, including proteomic signatures characterized through mass spectrometry, are ongoing to improve diagnostic precision and disease monitoring [31].

### 4.2 Genetic and Transcriptomic Signatures

Recent preliminary studies suggest that the inflammatory milieu in iMCD may represent a paraneoplastic process driven by an underlying clonal or neoplastic event, akin to the hypothesis proposed for POEMS syndrome [32, 33, 34]. This proposition is supported by intermittent cytogenetic abnormalities, including recurrent chromosome 7 abnormalities, as well as somatic variants in genes involved in the MAPK pathway (e.g., *PTPRR*, *ERBB2*, *FAS*, *STK3*, and *TGFBR2*) and epigenetic regulation (*SETD1A*, *ASH1L*, *DOT1L*, *KMT2E*, and *DNMT3A*) [34, 35, 36]. Moreover, recurrent variants in *NCOA4* [7, 35] and copy number alterations in *ETS*, *PTPN6*, *TGFBR2*, and *TUSC3* [32, 36] further substantiate this hypothesis. Notably, germline homozygous variants in *NCOA4* and heterozygous variants in tumor necrosis factor receptor‐associated factor (TRAF) have been identified in affected identical twins with distinct clinical phenotypes, highlighting the potential interplay between genetic predisposition and unidentified environmental factors [7].

Several groups have attempted to characterize the transcriptome of MCD, searching for the cell type(s) responsible for the known elevation in laboratory biomarkers IL‐6, VEGF, CXCL13, and others. An initial assessment of the transcriptome of MCD performed using targeted RNA‐sequencing (RNAseq) found upregulation of CCL21, a marker cytokine for fibroblastic reticular cells (FRCs), and IL‐6 pathway genes (*IL‐6*, *IL6ST*, *OSMR*, and *LIFR*) in MCD (*n* = 8) compared to UCD (*n* = 21) and control (*n* = 35) lymph nodes [37]. In addition, complement pathway genes were also upregulated in both MCD and UCD compared to controls. By RNA in situ hybridization, IL‐6 localized to interfollicular lymph node areas near CD31‐positive endothelial or lymphatic structures, while increased VEGF was distributed diffusely across the interfollicular area. Bulk whole exome sequencing and transcriptomic profiling confirmed upregulation of complement cascade elements, in addition to upregulation of VEGFR1 ligand, placental growth factor (PGF), and CXCL13 in iMCD, with IHC showing C4d upregulation within abnormal germinal centers [38].

More recently, the first single‐cell transcriptomic analysis of iMCD has been performed [7]. Using both dissociated single cells and in situ/spatial transcriptomics of one lymph node from a patient with iMCD, IL‐6 pathway genes were found to be predominantly expressed in endothelial and FRCs. Immunofluorescent co‐localization of IL‐6 and IL6ST with CD31 (endothelial marker) or CCL21 (FRC marker) corroborated the single‐cell sequencing findings [7]. These results strongly suggest that the lymph node microenvironment itself, particularly the stromal compartment, is driving the hypercytokinemia in iMCD. In Figure 2, we present an integrative schematic of the iMCD lymph node microenvironment, highlighting the histologic compartments and key cytokine‐producing cells (e.g., FRCs, endothelial cells) along with the major cytokines (IL‐6, VEGF) implicated in disease pathogenesis. The model illustrates how dysregulated stromal‐vascular‐immune cell interactions may lead to the characteristic histopathological and clinical features of iMCD.

## 5 Future Directions in the Diagnosis of iMCD

Significant opportunities exist to improve how we approach iMCD through personalized diagnosis, prognosis, and treatment. Research efforts must increasingly focus on dissecting disease pathophysiology at the single‐cell and spatial level, while also building integrated computational tools that enhance diagnostic reproducibility and therapeutic targeting.

### 5.1 Bringing Personalized Medicine to iMCD

The rarity of this disease process has thus far made it difficult to glean insights beyond that of the cohort or population level. However, as our patient population expands, our electronic health records mature, and our skills in real‐world data analysis develop, we may soon be able to more readily separate the individual from the population, with the goal of providing tailored inferences of the prognostic and therapeutic significance of each clinical feature in a new patient's presentation.

Personalized iMCD management may include integrating high‐resolution molecular tools such as targeted sequencing, RNA expression panels, or proteomic signatures into the diagnostic workflow. For instance, detection of an endothelial IL‐6/FRC‐VEGF signature via multiplex IHC could stratify patients by response to IL‐6 blockade. These tools will require close collaboration between test developers, regulatory authorities, and clinical laboratories to enable routine implementation. In parallel, the development of assays to identify patients unlikely to respond to first‐line therapies (e.g., those with a TNF‐dominant signature) could guide escalation to alternative immunomodulatory regimens. The “personalization” of iMCD diagnosis and treatment may take the form of expanding our diagnostic toolkit to include more genomic, transcriptomic, or proteomic data. If so, clinicians, researchers, and manufacturers will need to work in tandem to provide such testing for clinical use. In the more immediate future, the development of diagnostic tools to identify patients for whom standard‐of‐care is unlikely to succeed may provide significant value [39].

The expansion of real‐world datasets and larger clinical trial cohorts may also allow for a more nuanced approach to the diagnosis of iMCD. The complex pathophysiology underlying iMCD makes it challenging to diagnose with high positive and negative predictive values. However, as we develop better tools for data mining and data science, we may discover more effective ways to rule in or rule out the disease and its many alternatives in the differential diagnosis.

### 5.2 Promising Research Areas

Continued exploration in molecular biology, histopathology, and clinical biomarkers is essential for the evolution of iMCD diagnostics. One highly promising area of active research focuses on the cellular microenvironment surrounding pathological tissue sections, as the current data suggest that iMCD is likely a disease originating from lymph node stromal cells. Thus, by developing a more complete understanding of the genomic and cellular properties of normal and abnormal lymph node stromal cells, we can more readily develop diagnostic and therapeutic tools to leverage those differences.

One promising such area is the tandem application of dissociated and in situ/spatial single‐cell transcriptomics technologies with multiplex proteomic analysis (e.g., CODEX), allowing for the detailed examination of cellular composition, cellular neighborhoods, cell states, and possible genetic alterations on a single‐cell level. Quite recently, the first combinatory assessment of single‐cell transcriptomics with multiplex proteomic analysis of 3 cases of iMCD and one case of HHV‐8‐MCD has been published by Smith et al. [40]. This study demonstrated that the cellular composition in MCD shows an increased proportion of proliferating PCs, cytotoxic and memory T cells, monocytes/macrophages, endothelial/lymphatic cells, and FRCs compared to two noninfectious controls. While stromal cells are likely underrepresented in their scRNAseq data due to pre‐analytical dissociation loss, multiple stromal cell subtypes were identified, with IL‐6 genes being upregulated in T‐zone reticular cells (TRCs), B‐zone reticular cells (BRCs), and lymphatic endothelial cells (LECs). VEGF expression was enriched in TRCs, perivascular reticular cells (PRCs), and FDCs. RNA‐ISH co‐localization of IL‐6 and VEGF with PDGFR (FRC marker) or CXCL13 (FDC marker) corroborated the single‐cell sequencing findings. Future studies using tandem application of dissociated and in situ/spatial single‐cell transcriptomics technologies with multiplex proteomic analysis may benefit from attempting to enrich lymph node stromal cells to provide a more comprehensive and representative transcriptomic assessment of lymph node stromal cells. In addition, the development of a proteomic multiplex antibody panel with extensive stromal cell markers would also likely be required to more accurately characterize the “cell state” of these cell types and how they interact with neighboring immune cells. Overall, comprehensive genomic and proteomic profiling could significantly improve diagnostic accuracy, advance understanding of etiology and pathogenesis, and better inform patient treatment.

Beyond the transcriptome, authors have also explored the inflammatory profiles and their disturbances in patients with iMCD [41] as well as the proteomic‐level predictive power of a subset of potential biomarkers [42]. The interplay between IL‐6‐mediated cellular responses, the PI3K/AKT/mTOR pathway, and the immediate inflammatory makeup of affected bone marrow and other cellular immune mechanisms, such as a tumor necrosis factor signaling pathway, may provide actionable drug targets for future clinical trials [30, 43].

### 5.3 Digital Pathology and Machine Learning

As the digital infrastructures of our health systems continue to mature, we are presented with opportunities to leverage the tools of machine learning and artificial intelligence within our clinical workflows. Digital pathology involves the digitization of histological slides, enabling high‐resolution visualization and analysis of tissue samples. When combined with powerful new machine learning approaches, it is possible to develop algorithms that can recognize complex patterns and features and diagnose iMCD more quickly and accurately. It may also allow the identification of subtle prognostic or therapeutic nuances.

Importantly, we can learn from analogies in related diseases. In the field of lymphoma, deep learning algorithms have been trained on digitized biopsy images to classify lymphoma subtypes and even predict underlying molecular alterations [44, 45]. For instance, AI models have distinguished between different non‐Hodgkin lymphoma subtypes with high accuracy, a task that traditionally requires expert hematopathologists. Such successes suggest that a similar approach could be applied to CD, training a model to differentiate iMCD lymph node morphology from reactive hyperplasia or other mimics (e.g., lupus lymphadenitis, or HHV‐8‐associated MCD) based on patterns of follicles, vascularity, and plasmacytosis. In addition, fundamental image analysis tasks like cell segmentation and quantification, already employed in digital analysis of lymphoma histology, could be leveraged to objectively measure features in iMCD nodes. For example, an algorithm could count PCs in interfollicular regions or measure the radius of mantle zones around follicles, or count vascular structures, providing an unbiased quantification of “plasmacytic” versus “hypervascular” features.

The incorporation of multiplex IHC and immunofluorescence assays could allow for the simultaneous detection of multiple antigens, including IL‐6, VEGF, FDC markers, and have machine learning integrate this information to evaluate if a case has an FDC IL‐6 signature typical of iMCD; improving diagnostic accuracy [40].

Beyond diagnosis, digital pathology can aid in risk stratification. In pediatric nodular lymphocyte predominant Hodgkin lymphoma, for example, image analysis on digital slides was used to correlate certain histologic patterns with treatment response [46]. Analogously, in iMCD, one might find that certain quantified features (like degree of vascularization or extent of follicular dendritic cell disruption) correlate with the likelihood of relapse or refractoriness to IL‐6 therapy. These tools could provide decision support by flagging subtle findings standardizing interpretations across pathologists. Ultimately, integrating digital pathology, spatial “omics” and AI‐driven analytics holds great promise for refining diagnostic and prognostic algorithms in iMCD.

Achieving these goals would require substantial collaboration across many institutions, with subject‐matter experts in hematopathology, computer science, database administration, clinical informatics, and more contributing their unique perspectives and expertise. We envision that, as a part of future multidisciplinary workflows, a diagnosed iMCD patient's data could be fed into a predictive platform that suggests optimal therapy (for instance, identifying a patient with a prominent TNF gene signature who might benefit from anti‐TNF therapy versus anti‐IL‐6 treatment). The development of a common data registry and repository for iMCD would be a crucial first step in this process, though requiring substantial institutional efforts to aggregate, de‐identify, and standardize the meaningful data elements necessary for building and evaluating these models.

## 6 Discussion

In the past decade, the field of iMCD has evolved from a state of diagnostic uncertainty with nonspecific clinical presentations and reliance on exclusionary tactics into a multidisciplinary, evidence‐based domain that integrates clinical, histopathological, molecular, and computational advances. The adoption of international consensus criteria, informed by comprehensive patient registries and collaborative networks, has provided a more uniform diagnostic foundation and fostered rapid recognition of disease subtypes like TAFRO. Complementary improvements in imaging modalities, histological techniques, and targeted biomarker profiling have further enhanced diagnostic accuracy, ushering in a new era of precision medicine.

Molecular and genomic insights are now reshaping our understanding of iMCD pathobiology, revealing complex cytokine networks, potential paraneoplastic drivers, and immunological aberrations that may underlie distinct clinical phenotypes. The integration of digital pathology, spatial omics, and AI‐driven analytics holds the promise of refining diagnostic algorithms even further, aiding in early detection, prognostication, and therapeutic decision‐making. Though many questions remain, particularly regarding refractory disease states, molecular heterogeneity, and long‐term outcomes, the trajectory of iMCD research is one of increasing clarity, consensus, and sophistication. As we move forward, leveraging novel tools and expanding international collaboration will be essential for translating these advances into tangible improvements in patient care.

## Conflicts of Interest

The authors declare no conflicts of interest.

## Supporting information


**Data S1:** Supporting Information.

**Table S1:** Supporting Information.

## Data Availability

This review article does not include any original datasets. No new data were generated or analyzed in support of this publication.

## References

[1] B. Castleman and V. W. Towne , “Case Records of the Massachusetts General Hospital; Weekly Clinicopathological Exercises; Case No. 40351,” New England Journal of Medicine 251, no. 10 (1954): 396–400. 13194083 10.1056/NEJM195409022511008

[2] B. Castleman , L. Iverson , and V. P. Menendez , “Localized Mediastinal Lymphnode Hyperplasia Resembling Thymoma,” Cancer 9, no. 4 (1956): 822–830. 13356266 10.1002/1097-0142(195607/08)9:4<822::aid-cncr2820090430>3.0.co;2-4

[3] D. C. Fajgenbaum , T. S. Uldrick , A. Bagg , et al., “International, Evidence‐Based Consensus Diagnostic Criteria for HHV‐8‐Negative/Idiopathic Multicentric Castleman Disease,” Blood 129, no. 12 (2017): 1646–1657. 28087540 10.1182/blood-2016-10-746933 PMC5364342

[4] D. C. Fajgenbaum , F. van Rhee , and C. S. Nabel , “HHV‐8‐Negative, Idiopathic Multicentric Castleman Disease: Novel Insights Into Biology, Pathogenesis, and Therapy,” Blood 123, no. 19 (2014): 2924–2933. 24622327 10.1182/blood-2013-12-545087

[5] P. Uciechowski and W. C. M. Dempke , “Interleukin‐6: A Masterplayer in the Cytokine Network,” Oncology 98, no. 3 (2020): 131–137. 31958792 10.1159/000505099

[6] S. K. Pierson , S. Shenoy , A. B. Oromendia , et al., “Discovery and Validation of a Novel Subgroup and Therapeutic Target in Idiopathic Multicentric Castleman Disease,” Blood Advances 5, no. 17 (2021): 3445–3456. 34438448 10.1182/bloodadvances.2020004016 PMC8525223

[7] J. Y. Chan , J. W. Loh , J. Q. Lim , et al., “Single‐Cell Landscape of Idiopathic Multicentric Castleman Disease in Identical Twins,” Blood 143, no. 18 (2024): 1837–1844. 38170173 10.1182/blood.2023021992

[8] A. R. Keller , L. Hochholzer , and B. Castleman , “Hyaline‐Vascular and Plasma‐Cell Types of Giant Lymph Node Hyperplasia of the Mediastinum and Other Locations,” Cancer 29, no. 3 (1972): 670–683. 4551306 10.1002/1097-0142(197203)29:3<670::aid-cncr2820290321>3.0.co;2-#

[9] G. Frizzera , P. M. Banks , G. Massarelli , and J. Rosai , “A Systemic Lymphoproliferative Disorder With Morphologic Features of Castleman's Disease. Pathological Findings in 15 Patients,” American Journal of Surgical Pathology 7, no. 3 (1983): 211–231. 6837832 10.1097/00000478-198304000-00001

[10] R. T. Miller , K. Mukai , P. M. Banks , and G. Frizzera , “Systemic Lymphoproliferative Disorder With Morphologic Features of Castleman's Disease. Immunoperoxidase Study of Cytoplasmic Immunoglobulins,” Archives of Pathology & Laboratory Medicine 108, no. 8 (1984): 626–630. 6204622

[11] S. Mori , N. Mohri , T. Uchida , and T. Shimamine , “Idiopathic Plasmacytic Lymphadenopathy With Polyclonal Hyperimmunoglobulinemia: A Syndrome Related to Giant Lymph Node Hyperplasia of Plasma Cell Type,” Journal of the Japanese Society for Lymphoreticular Tissue Research 20 (1980): 55–65.

[12] K. T. Chen , “Multicentric Castleman's Disease and Kaposi's Sarcoma,” American Journal of Surgical Pathology 8, no. 4 (1984): 287–294. 6711739 10.1097/00000478-198404000-00006

[13] N. A. Lachant , N. C. Sun , L. A. Leong , R. S. Oseas , and H. E. Prince , “Multicentric Angiofollicular Lymph Node Hyperplasia (Castleman's Disease) Followed by Kaposi's Sarcoma in Two Homosexual Males With the Acquired Immunodeficiency Syndrome (AIDS),” American Journal of Clinical Pathology 83, no. 1 (1985): 27–33. 3871303 10.1093/ajcp/83.1.27

[14] A. M. Rywlin , L. Rosen , and B. Cabello , “Coexistence of Castleman's Disease and Kaposi's Sarcoma. Report of a Case and a Speculation,” American Journal of Dermatopathology 5, no. 3 (1983): 277–281. 6625119 10.1097/00000372-198306000-00015

[15] J. Soulier , L. Grollet , E. Oksenhendler , et al., “Kaposi's Sarcoma‐Associated Herpesvirus‐Like DNA Sequences in Multicentric Castleman's Disease,” Blood 86, no. 4 (1995): 1276–1280. 7632932

[16] R. Warsame , U. Yanamandra , and P. Kapoor , “POEMS Syndrome: An Enigma,” Current Hematologic Malignancy Reports 12, no. 2 (2017): 85–95. 28299525 10.1007/s11899-017-0367-0

[17] N. Kurose , C. Futatsuya , K. I. Mizutani , et al., “The Clinicopathological Comparison Among Nodal Cases of Idiopathic Multicentric Castleman Disease With and Without TAFRO Syndrome,” Human Pathology 77 (2018): 130–138. 29684500 10.1016/j.humpath.2018.04.001

[18] K. Takai , K. Nikkuni , H. Shibuya , and H. Hashidate , “Thrombocytopenia With Mild Bone Marrow Fibrosis Accompanied by Fever, Pleural Effusion, Ascites and Hepatosplenomegaly,” Rinshō Ketsueki 51, no. 5 (2010): 320–325. 20534952

[19] R. Sumiyoshi , T. Koga , and A. Kawakami , “Biomarkers and Signaling Pathways Implicated in the Pathogenesis of Idiopathic Multicentric Castleman Disease/Thrombocytopenia, Anasarca, Fever, Reticulin Fibrosis, Renal Insufficiency, and Organomegaly (TAFRO) Syndrome,” Biomedicine 12, no. 6 (2024): 1141. 10.3390/biomedicines12061141 PMC11200392 38927348

[20] H. Kawabata , K. Takai , M. Kojima , et al., “Castleman‐Kojima Disease (TAFRO Syndrome): A Novel Systemic Inflammatory Disease Characterized by a Constellation of Symptoms, Namely, Thrombocytopenia, Ascites (Anasarca), Microcytic Anemia, Myelofibrosis, Renal Dysfunction, and Organomegaly: A Status Report and Summary of Fukushima (6 June, 2012) and Nagoya Meetings (22 September, 2012),” Journal of Clinical and Experimental Hematology 53, no. 1 (2013): 57–61. 10.3960/jslrt.53.57 23801135

[21] R. Alaggio , C. Amador , I. Anagnostopoulos , et al., “The 5th Edition of the World Health Organization Classification of Haematolymphoid Tumours: Lymphoid Neoplasms,” Leukemia 36, no. 7 (2022): 1720–1748. 35732829 10.1038/s41375-022-01620-2 PMC9214472

[22] R. Alaggio , C. Amador , I. Anagnostopoulos , et al., “Correction: “The 5th Edition of the World Health Organization Classification of Haematolymphoid Tumours: Lymphoid Neoplasms” *Leukemia*. 2022 Jul;36(7):1720–1748,” Leukemia 37, no. 9 (2023): 1944–1951. 35732829 10.1038/s41375-022-01620-2 PMC9214472

[23] E. Belyaeva , A. Rubenstein , S. K. Pierson , et al., “Bone Marrow Findings of Idiopathic Multicentric Castleman Disease: A Histopathologic Analysis and Systematic Literature Review,” Hematological Oncology 40, no. 2 (2022): 191–201. 35104370 10.1002/hon.2969 PMC9547646

[24] G. Srkalovic , I. Marijanovic , M. B. Srkalovic , and D. C. Fajgenbaum , “TAFRO Syndrome: New Subtype of Idiopathic Multicentric Castleman Disease,” Bosnian Journal of Basic Medical Sciences 17, no. 2 (2017): 81–84. 28135567 10.17305/bjbms.2017.1930 PMC5474112

[25] F. van Rhee , A. Rosenthal , K. Kanhai , et al., “Siltuximab Is Associated With Improved Progression‐Free Survival in Idiopathic Multicentric Castleman Disease,” Blood Advances 6, no. 16 (2022): 4773–4781. 35793409 10.1182/bloodadvances.2022007112 PMC9631655

[26] C. Jitaru , A. Symeonidis , S. Badelita , et al., “Siltuximab in Idiopathic Multicentric Castleman Disease: Real‐World Experience,” Journal of Hematology 13, no. 5 (2024): 207–215. 39493599 10.14740/jh1343 PMC11526577

[27] N. Nishimoto , Y. Kanakura , K. Aozasa , et al., “Humanized Anti‐Interleukin‐6 Receptor Antibody Treatment of Multicentric Castleman Disease,” Blood 106, no. 8 (2005): 2627–2632. 15998837 10.1182/blood-2004-12-4602

[28] S. K. Pierson , M. S. Lim , G. Srkalovic , et al., “Treatment Consistent With Idiopathic Multicentric Castleman Disease Guidelines Is Associated With Improved Outcomes,” Blood Advances 7, no. 21 (2023): 6652–6664. 37656441 10.1182/bloodadvances.2023010745 PMC10637880

[29] L. Zhang , A. L. Zhao , M. H. Duan , et al., “Phase 2 Study Using Oral Thalidomide‐Cyclophosphamide‐Prednisone for Idiopathic Multicentric Castleman Disease,” Blood 133, no. 16 (2019): 1720–1728. 30760451 10.1182/blood-2018-11-884577

[30] D. C. Fajgenbaum , R. A. Langan , A. S. Japp , et al., “Identifying and Targeting Pathogenic PI3K/AKT/mTOR Signaling in IL‐6‐Blockade‐Refractory Idiopathic Multicentric Castleman Disease,” Journal of Clinical Investigation 129, no. 10 (2019): 4451–4463. 31408438 10.1172/JCI126091 PMC6763254

[31] Y. Park , M. J. Kim , Y. Choi , et al., “Role of Mass Spectrometry‐Based Serum Proteomics Signatures in Predicting Clinical Outcomes and Toxicity in Patients With Cancer Treated With Immunotherapy,” Journal for Immunotherapy of Cancer 10, no. 3 (2022): e003566. 35347071 10.1136/jitc-2021-003566 PMC8961104

[32] A. Butzmann , J. Kumar , K. Sridhar , S. Gollapudi , and R. S. Ohgami , “A Review of Genetic Abnormalities in Unicentric and Multicentric Castleman Disease,” Biology 10, no. 4 (2021): 251. 33804823 10.3390/biology10040251 PMC8063830

[33] A. Carbone , M. Borok , B. Damania , et al., “Castleman Disease,” Nature Reviews Disease Primers 7, no. 1 (2021): 84. 10.1038/s41572-021-00317-7 PMC9584164 34824298

[34] A. Gonzalez Garcia , J. Fernandez‐Martin , and A. Robles Marhuenda , “Idiopathic Multicentric Castleman Disease and Associated Autoimmune and Autoinflammatory Conditions: Practical Guidance for Diagnosis,” Rheumatology 62, no. 4 (2023): 1426–1435. 35997567 10.1093/rheumatology/keac481 PMC10070070

[35] L. You , Q. Lin , J. Zhao , F. Shi , K. H. Young , and W. Qian , “Whole‐Exome Sequencing Identifies Novel Somatic Alterations Associated With Outcomes in Idiopathic Multicentric Castleman Disease,” British Journal of Haematology 188, no. 5 (2020): e64–e67. 31863597 10.1111/bjh.16330

[36] A. Nagy , A. Bhaduri , N. Shahmarvand , et al., “Next‐Generation Sequencing of Idiopathic Multicentric and Unicentric Castleman Disease and Follicular Dendritic Cell Sarcomas,” Blood Advances 2, no. 5 (2018): 481–491. 29496669 10.1182/bloodadvances.2017009654 PMC5851414

[37] A. Wing , J. Xu , W. Meng , et al., “Transcriptome and Unique Cytokine Microenvironment of Castleman Disease,” Modern Pathology 35, no. 4 (2022): 451–461. 34686774 10.1038/s41379-021-00950-3 PMC9272352

[38] P. Horna , R. L. King , D. Jevremovic , D. C. Fajgenbaum , and A. Dispenzieri , “The Lymph Node Transcriptome of Unicentric and Idiopathic Multicentric Castleman Disease,” Haematologica 108, no. 1 (2023): 207–218. 35484648 10.3324/haematol.2021.280370 PMC9827154

[39] S. K. Pierson , Y. Ren , J. Khor , et al., “Natural History Study of Idiopathic Multicentric Castleman Disease Identifies Effective Treatments for a Large Proportion of Patients but Treatment‐Refractory Patients Remain,” Blood 134 (2019): 1540.

[40] D. Smith , A. Eichinger , A. Rech , et al., “Spatial and Single Cell Mapping of Castleman Disease Reveals Key Stromal Cell Types and Cytokine Pathways,” Nature Communications 16 (2025): 6009. 10.1038/s41467-025-61214-1 PMC12217310 40593805

[41] R. L. Pai , A. S. Japp , M. Gonzalez , et al., “Type I IFN Response Associated With mTOR Activation in the TAFRO Subtype of Idiopathic Multicentric Castleman Disease,” JCI Insight 5, no. 9 (2020): e135031. 32376796 10.1172/jci.insight.135031 PMC7253017

[42] R. Sumiyoshi , T. Koga , and A. Kawakami , “Serum Proteomics Reveals Insulin‐Like Growth Factor Binding Proteins‐1 as Biomarkers for Idiopathic Multicentric Castleman's Disease,” Annals of the Rheumatic Diseases 80 (2021): 959.

[43] M. D. Mumau , M. V. Gonzalez , C. Ma , et al., “Identifying and Targeting TNF Signaling in Idiopathic Multicentric Castleman's Disease,” New England Journal of Medicine 392, no. 6 (2025): 616–618. 39908436 10.1056/NEJMc2412494 PMC11801236

[44] H. E. Achi , T. Belousova , L. Chen , et al., “Automated Diagnosis of Lymphoma With Digital Pathology Images Using Deep Learning,” Annals of Clinical and Laboratory Science 49, no. 2 (2019): 153–160. 31028058

[45] G. Steinbuss , M. Kriegsmann , C. Zgorzelski , et al., “Deep Learning for the Classification of Non‐Hodgkin Lymphoma on Histopathological Images,” Cancers 13, no. 10 (2021): 2419. 34067726 10.3390/cancers13102419 PMC8156071

[46] S. Sereda , A. Shankar , L. Weber , et al., “Digital Pathology in Pediatric Nodular Lymphocyte‐Predominant Hodgkin Lymphoma: Correlation With Treatment Response,” Blood Advances 7, no. 20 (2023): 6285–6289. 37611165 10.1182/bloodadvances.2023010652 PMC10589766

