# Supplementary material for: The Evolution and Recent Advances in Diagnostic Criteria for Idiopathic Multicentric Castleman Disease
Source: Am J Hematol. 2025 Aug 21;100(11):2064–73. doi: 10.1002/ajh.70039 (PMC12516665; doi:10.1002/ajh.70039)

Review of the Evolution of Diagnostic Criteria for Idiopathic Multicentric Castleman Disease and Recent Advances

Supplementary data:

Supplemental Figure 1: A) Future research projection chart. A PubMed search on iMCD articles demonstrates a ~30% annual growth in publication numbers in recent years, with predicted values for 2025 to 2027. **B) Key milestones in Castleman disease research, diagnosis, and therapy in the 2000s**. These include the establishment of the Castleman Disease Collaborative Network (CDCN), advancements in diagnostic criteria, and ongoing therapeutic trials and research. CDCN, Castleman Disease Collaborative Network; AI, artificial intelligence; BCD regimen, a combination of bortezomib, cyclophosphamide, and dexamethasone; iMCD, idiopathic multicentric Castleman disease.

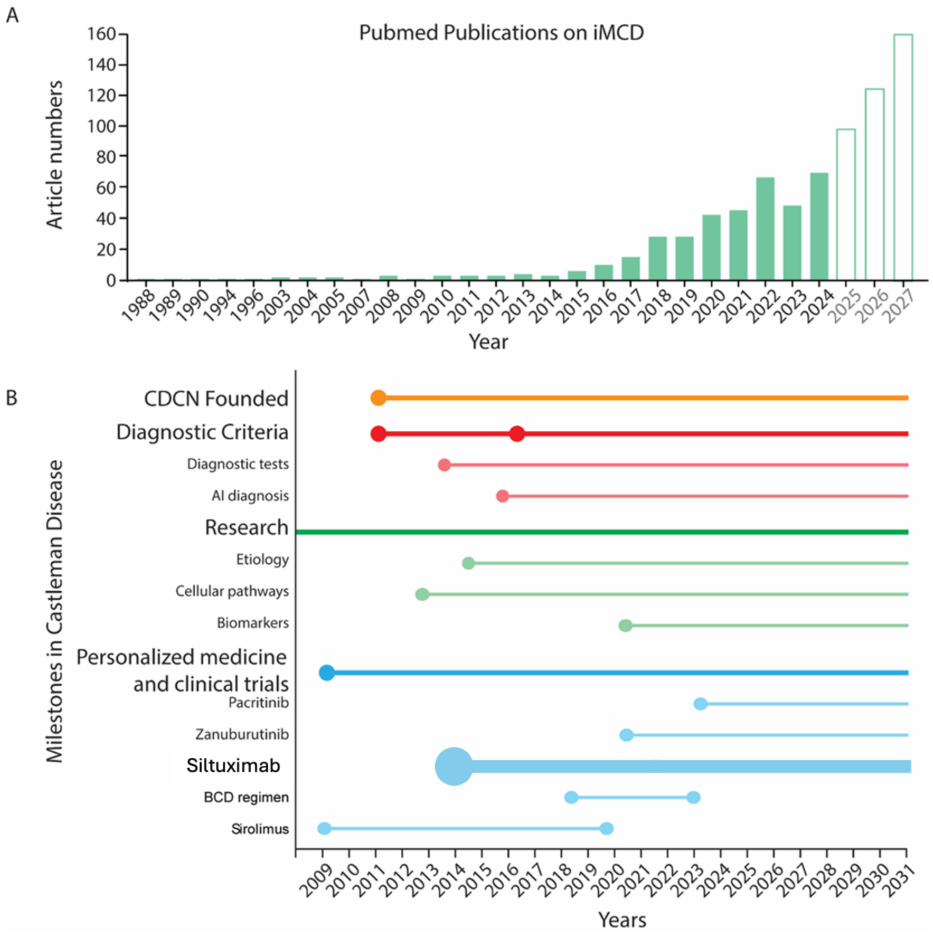

Supplement: Supplementary file 1 — Data S1: Supporting Information. [file AJH-100-2064-s002.pdf]
