# Supplementary material for: The Evolution and Recent Advances in Diagnostic Criteria for Idiopathic Multicentric Castleman Disease
Source: Am J Hematol. 2025 Aug 21;100(11):2064–73. doi: 10.1002/ajh.70039 (PMC12516665; doi:10.1002/ajh.70039)
Supplement: Supplementary file 2 — Table S1: Supporting Information. [file AJH-100-2064-s001.docx]

**Supplementary Table 1: Terminology**

| **Terms** | **Definition** |
| --- | --- |
| Anasarca | Severe generalized edema caused by fluid retention; a hallmark feature of TAFRO syndrome in iMCD. |
| Castleman Disease Collaborative Network (CDCN) | A global organization dedicated to advancing research, building consensus, and improving the understanding and treatment of Castleman disease. |
| CRP (C-reactive protein) | An acute-phase reactant synthesized in the liver in response to IL-6; elevated levels are a marker of systemic inflammation in iMCD. |
| CXCL13 | A chemokine critical for B-cell recruitment and germinal center formation; elevated levels are a hallmark of immune activation in iMCD. |
| Cytokine Storm | A hyperinflammatory state marked by excessive cytokine release, particularly IL-6, leads to systemic inflammation and multi-organ dysfunction. |
| Digital Pathology | The application of digital imaging, artificial intelligence, and computational algorithms to analyze histological slides, enhancing diagnostic precision and reproducibility. |
| Epigenetic Regulation | Mechanisms that alter gene expression without changing the DNA sequence and disruptions in epigenetic regulation (e.g., variants in *SETD1A* and *KMT2E*) are linked to iMCD pathogenesis. |
| Fibroblastic Reticular Cells (FRCs) | Stromal cells responsible for lymph node structural integrity and immune cell migration that are implicated in the production of IL-6 and VEGF in iMCD. |
| Follicular Dendritic Cells (FDCs) | Specialized stromal cells within lymphoid follicles that support B-cell maturation and immune memory; in iMCD, these cells often show prominence or network disruption. |
| Hypervascular (HV) Subtype | A histological variant of iMCD, characterized by regressed germinal centers, prominent follicular dendritic cell networks, and hyalinized blood vessels penetrating germinal centers. |
| Idiopathic Multicentric Castleman Disease (iMCD) | A rare, systemic cytokine driven disorder with no known viral etiology, characterized by systemic inflammation, lymphadenopathy, organ dysfunction, and abnormal lymph node architecture. |
| IgG4-Related Disease | An immune-mediated fibroinflammatory disorder that mimics iMCD, characterized by dense IgG4-positive plasma cell infiltrates, storiform fibrosis, and elevated serum IgG4 levels. |
| Interleukin-6 (IL-6) | A pro-inflammatory cytokine central to iMCD pathogenesis that drives systemic inflammation, B-cell proliferation, and acute-phase responses such as increased CRP and anemia. |
| Mixed Subtype | A histological variant of iMCD exhibiting features of both HV and PC subtypes, reflecting the spectrum of morphological changes within the disease. |
| Multiplex Immunohistochemistry | A technique that allows simultaneous detection of multiple antigens on a single tissue section; this can be used to characterize FDC networks and plasma cell clonality in iMCD. |
| Organomegaly | Abnormal enlargement of organs such as the spleen or liver; this is is commonly seen in iMCD due to systemic inflammation and immune dysregulation. |
| Paraneoplastic Syndrome | A disorder arising from immune responses to tumors, proposed as a potential underlying mechanism in certain cases of iMCD due to findings of clonal processes or genomic alterations. |
| Plasmacytic (PC) Subtype | A histological variant of iMCD with hyperplastic germinal centers, interfollicular plasmacytosis, and increased polyclonal plasma cells reflecting systemic inflammation. |
| Polyclonal Hypergammaglobulinemia | Elevated levels of multiple immunoglobulin subclasses (e.g., IgG, IgA) without clonality; often can be seen due to widespread immune activation in iMCD. |
| Reticulin Fibrosis | The thickening of reticulin fibers within the bone marrow; this is observed in TAFRO syndrome, reflects stromal cell activation and cytokine-driven changes. |
| Siltuximab | A monoclonal antibody therapy that targets and neutralizes IL-6, improving symptoms and outcomes in iMCD patients. |
| Single-cell Sequencing | A genomic technology used to analyze the gene expression of individual cells; in iMCD, it has identified cellular subsets, such as fibroblastic reticular cells, that contribute to disease pathology. |
| Spatial Transcriptomics | A technology combining spatial mapping of tissue architecture with gene expression analysis; in iMCD-affected tissues, it has revealed the molecular landscape and cytokine-producing cells. |
| TAFRO Syndrome | A severe clinical subtype of iMCD characterized by thrombocytopenia, anasarca, fever, reticulin fibrosis (or renal dysfunction), and organomegaly, often associated with hypervascular histopathology. |
| Thrombocytopenia | A reduction in platelet count; it is commonly seen in TAFRO syndrome and is reflective of bone marrow dysfunction or cytokine-mediated platelet destruction. |
| VEGF (Vascular Endothelial Growth Factor) | A cytokine promoting blood vessel growth and permeability, often elevated in iMCD, that contributes to vascular abnormalities and systemic inflammation. |
